# Interventions to prevent obesity in the first 1000 days: scoping review

**DOI:** 10.15649/cuidarte.3558

**Published:** 2024-09-01

**Authors:** Paola Alexandra Zepeda-Ríos, Velia Margarita Cárdenas-Villarreal, Danilo Castro-Sifuentes, Milton Carlos Guevara-Valtier

**Affiliations:** 1 School of Nursing, Universidad Autónoma de Nuevo León. Monterrey, Mexico. E-mail: paola.zepedar@uanl.edu.mx Universidad Autónoma de Nuevo León School of Nursing Universidad Autónoma de Nuevo León Monterrey Mexico paola.zepedar@uanl.edu.mx; 2 School of Nursing, Universidad Autónoma de Nuevo León. Monterrey, Mexico. E-mail: velia.cardenasvl@uanl.edu.mx Universidad Autónoma de Nuevo León School of Nursing Universidad Autónoma de Nuevo León Monterrey Mexico velia.cardenasvl@uanl.edu.mx; 3 School of Nursing, Universidad Autónoma de Nuevo León. Monterrey, Mexico. E-mail danilo.castros@uanl.edu.mx Universidad Autónoma de Nuevo León School of Nursing Universidad Autónoma de Nuevo León Monterrey Mexico danilo.castros@uanl.edu.mx; 4 School of Nursing, Universidad Autónoma de Nuevo León. Monterrey, Mexico. E-mail milton.guevaravlt@uanl.edu.mx Universidad Autónoma de Nuevo León School of Nursing Universidad Autónoma de Nuevo León Monterrey Mexico milton.guevaravlt@uanl.edu.mx

**Keywords:** Childhood Obesity, Overweight, Infant, Prevention, Obesidad Infantil, Sobrepeso, Lactante, Prevención, Obesidade Infantil, Sobrepeso, Lactente, Prevenção

## Abstract

**Introduction:**

The global prevalence of childhood obesity is a public health challenge. Early intervention, including during pregnancy, is essential to prevent this health problem.

**Objective:**

Identify and characterize interventions during the first 1000 days of life that effectively prevent overweight and obesity.

**Materials and Methods:**

A scoping review was carried out following the methodology proposed by Arksey and O'Malley. SCOPUS, EBSCOHost and PubMed databases were searched to select relevant articles. The analysis focused on articles published between January 2012 and December 2022.

**Results:**

Fourteen articles with 10 interventions were included. These interventions were implemented in high-income countries and in racial and ethnic groups. Three interventions, involving 1013 women and their children, reported significant effects on preventing overweight and obesity at 18 and 24 months of the child's life.

**Discussion:**

The interventions were characterized as multi-component, educational and based on theories of behavior change, parenting and sensory feeding. They addressed risk factors such as breastfeeding, complementary feeding, physical activity and sleep. There is an increasing use of digital technology in their delivery.

**Conclusion:**

Promising results have been found for the prevention of obesity in the early years of life; therefore, implementation of interventions in low- and middle-income countries is of paramount importance.

## Resumen.

**Introducción:**

La prevalencia mundial de la obesidad infantil plantea un desafío para la salud pública. Para prevenir este problema de salud, es crucial intervenir tempranamente, incluso durante el embarazo.

**Objetivo:**

Identificar y caracterizar intervenciones realizadas durante los primeros 1000 días de vida que eviten de manera efectiva el sobrepeso y la obesidad.

**Materiales y Métodos:**

Se realizó una revisión de alcance siguiendo la metodología propuesta por Arksey y O'Malley. Se realizaron búsquedas en las bases de datos SCOPUS, EBSCOHost y PubMed para seleccionar artículos relevantes. El análisis se centró en artículos publicados entre enero de 2012 y diciembre de 2022.

**Resultados:**

Se incluyeron 14 artículos que integraban 10 intervenciones. Estas fueron implementadas en países de altos ingresos y con grupos raciales/étnicos. Tres intervenciones, conformadas por 1013 mujeres y sus hijos, reportaron efectos significativos en la prevención de sobrepeso y obesidad a los 18 meses y 24 meses de edad del niño.

**Discusión:**

Las intervenciones se caracterizaron por ser multicomponentes, educativas, basadas en teorías de cambio de comportamiento, crianza de los hijos y alimentación perceptiva. Abordaron factores de riesgo como la lactancia materna, alimentación complementaria, actividad física y el sueño. Existe una creciente incorporación de tecnología digital en su implementación.

**Conclusión:**

Se han observado resultados prometedores para la prevención de la obesidad en los primeros años de vida. Implementar intervenciones en países de bajos y medianos ingresos resulta primordial.

## Resumo.

**Introdução:**

A prevalência global da obesidade infantil representa um desafio para a saúde pública. Para prevenir este problema de saúde é fundamental intervir precocemente, mesmo durante a gravidez.

**Objetivo:**

Identificar e caracterizar intervenções realizadas durante os primeiros 1000 dias de vida que previnam eficazmente o excesso de peso e a obesidade.

**Materiais e Métodos:**

Foi realizada uma revisão de escopo seguindo a metodologia proposta por Arksey e O'Malley. As bases de dados SCOPUS, EBSCOHost e PubMed foram pesquisadas para selecionar artigos relevantes. A análise centrou-se em artigos publicados entre janeiro de 2012 e dezembro de 2022.

**Resultados:**

Foram incluídos 14 artigos que integraram 10 intervenções. Estas foram implementadas em países de rendimento elevado e com grupos raciais/étnicos. Três intervenções, compostas por 1.013 mulheres e seus filhos, relataram efeitos significativos na prevenção do sobrepeso e da obesidade aos 18 meses e aos 24 meses de idade da criança.

**Discussão:**

As intervenções caracterizaram-se como multicomponentes, educativas, baseadas em teorias de mudança de comportamento, parentalidade e alimentação responsiva. Abordaram fatores de risco como amamentação, alimentação complementar, atividade física e sono. Há uma crescente incorporação da tecnologia digital na sua implementação.

**Conclusão:**

Resultados promissores foram observados para a prevenção da obesidade nos primeiros anos de vida. A implementação de intervenções em países de baixo e médio rendimento é essencial.

## Introduction

Over the past four decades, the global prevalence of childhood obesity has increased exponentially^1^, with 37 million children under the age of five being overweight or obese^2^. Being overweight or obese in the early years of life has been shown to increase the risk of developing a number of early diseases such as asthma, coronary heart disease, type 2 diabetes mellitus, osteoarthritis, some cancers and others^3^^, ^^4^, and is responsible for 2.6 million deaths each year^5^. Preventing childhood obesity is therefore a priority^6^.

Early life is a critical period for the development of obesity. International child health organizations have developed guidelines and recommendations to prevent, control and reduce obesity during the first 1000 days^7^ - a crucial period of human development from conception to 2 years of age^8^. These recommendations aim to implement evidence-based interventions that comprehensively address key modifiable risk factors during the prenatal and postnatal stages to prevent childhood obesity^9^^, ^^10^. It is, therefore, important to identify the types of interventions that have been implemented to prevent childhood obesity in the first 1000 days and whether they have evidence of effectiveness.

Previous systematic reviews have assessed interventions in these stages and found promising results; however, the prenatal or postnatal stages have been considered indistinctly^11^^, ^^12^, so this review sought to assess only those interventions that considered both stages of development, as the evidence mentions the importance of considering the first 1000 days in a comprehensive way^9^. Therefore, the aim of this scoping review was to identify and characterize the interventions implemented in the first 1000 days (considering the prenatal and postnatal period together) for the prevention of overweight and obesity. The information gathered can serve as a guide for health care providers and researchers looking for opportunities to implement interventions aimed at preventing childhood obesity.

## Materials and Methods

This a registered scoping review^13^^, ^^14^, which used the 5 stages of Arksey and O'Malley's methodological framework^15^.

### Research question identification

What international evidence exists on interventions that address the first 1000 days of life with the objective of preventing overweight and obesity in children under two years of age?

What are the characteristics of interventions that have been effective in preventing overweight and obesity in children under two years of age at the international level?

### Relevant studies

In January 2023, a systematized search was conducted in the SCOPUS, EBSCOHost and PubMed databases, focused on the pre-established inclusion criteria and using the Medical Subject Headings (MeSH) vocabulary descriptors ofthe U.S. National Library of Medicine MeSH that included the words: *Infancy, infants, first 1000 days, preventive measures, prevention, control, intervention, overweight, obesity, body weight, pediatric obesity, randomized controlled trial, clinical trial randomized, systematic review.*

### Study selection

Criteria: Systematic reviews of randomized clinical trials and/or articles documenting a single randomized clinical trial; published between 1 January 2012 and 31 December 2022; interventions should consider the first 1000 days and focus on obesity prevention in children aged 0-2 years; with weight or BMI z-score by age as the primary outcome to homogenize and contrast studies; no language restriction. Non-systematic literature reviews, protocols, grey literature and pre-prints were excluded.

### Charting the data

The PRISMA-ScR extension framework was used to extract the information. The information was recorded independently by two of the authors using a form.

### Comparing, summarizing and communicating the results

The interventions were identified and summarized according to the patterns found in the extracted information^15^. The database was stored in Mendeley Data^16^.

### Ethical aspects

The review studies do not require ethics committee approval, but scientific rigor was essential in their realization.

## Results

**Figure 1:**
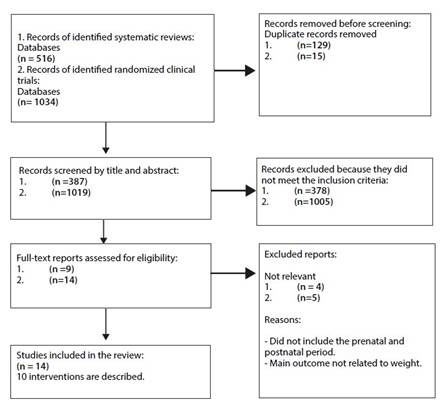
PRISMA-ScR flow diagram for identifying studies from databases and registries

For interventions reported in systematic reviews, a total of 387 references were identified after removing duplicates; 378 were excluded based on title and abstract, leaving a total of 9 articles to be assessed in full text. The search was supplemented with a strategy to identify randomized clinical trials of individual interventions, of which 5 were excluded because they did not meet the criteria, and the remaining 9 trials were considered eligible. The total number of articles included in both searches was 14, comprising 10 interventions^17^^, ^^30^. See Figure 1.

In order to answer the research questions, the corresponding articles were reviewed, and the results were categorized along three main dimensions: characteristics ofthe study population, characteristics of the intervention design, general characteristics and outcomes of effective interventions.

### Study population characteristics

All interventions were carried out in high-income countries, none in middle- or low-income countries. Participants were women aged over 18 years from low socioeconomic backgrounds. Four of them belonged to the Latino subcultural group, one to the African American group, and the rest were not specified (n=5). Half of the interventions (n=5) started when the mother was in the third quarter of pregnancy, and the rest in the second and third quarters (n=5). See Table 1.

**Table 1:** General characteristics of the study population, 2012-2022

Author/Year	Country of Residence	Mother's Age	Socioeconomic Status	Subcultural Group	Pregnancy Stage
Reifsnider et al., 2018^17^; McCormick et al., 2020^18^	United States	18 to 40 years old	Low	Latinas	Third quarter
Gross et al., 2016^19^; Messito et al., 2020^20^	United States	Over 18 years old	Low	Latinas/ Hispanics	Third quarter
Ordway et al., 2018^21^	United States	14 to 25 years old	Low	Different ethnic communities/Hispanics	Third quarter
Parat et al., 2018^22^	France	18 years and older	Low	Not specified	Equal to or less than 21 weeks of gestation
Fangupo 2015^23^; Taylor et al., 2017^24^ Taylor et al., 2018^25^	New Zealand	16 years and older	Not specified	Not specified	Before 34 weeks of gestation
Fiks et al., 2017^26^	United States	18 years and older	Low	Not specified	Third quarter
Thomson et al., 2018^27^	United States	18 years and older	Low	Not specified	Minimum 19 weeks of gestation
Wasser et al., 2020^28^	United States	18 to 39 years old	Low	Non-Hispanic African Americans	28 weeks of gestation
Wen et al., 2012^29^; 2015^30^	Australia	Over 16 years old	Low	Not specified	Between 24 and 34 weeks of gestation

Design characteristics of the interventions

Seven interventions used behavior change theory in their design, with social cognitive theory being the most commonly used. All interventions were face-to-face and delivered mainly by nurses and dietitians. The duration of the interventions ranged from 2 to 3 years.

Although all the interventions started in the prenatal period, only two of them addressed maternal factors, such as gestational weight gain. The others developed educational programs that focused on postnatal factors, such as infant nutrition.

Integration of physical activity and sleep was less common. Finally, the Starting Early Program (StEP) ^20^, Minding the Baby^21^, and Healthy Beginnings^29^^, ^^30^ interventions were found to have statistically significant outcomes related to infant weight. See Table 2.

**Table 2:** Design characteristics of the total number of interventions identified for the period 2012-2022

Author/Year	Theoretical Framework	Delivery	Duration	Environment and Number of Sessions	Attention to Intervention Group	Attention to Control Group	Primary Outcomes	Findings
Reifsnider et al., 2018^17^ McCormick et al., 2020^18^	Not specified	Face-to-face.	2 years	Home. 9 sessions: 36 weeks gestation, 2 weeks of age, 2, 4, 6, 9, 12, 18 and 24 months.	Child growth, breastfeeding, nutrition, physical activity and sleep.	Visits for measuring child growth and development.	Weight-for-length z-score of infants	Parents' education did not reduce childhood overweight.
Gross et al., 2016^19^ Messito et al., 2020^20^	Social Cognitive Theory	Face-to-face. Certified dietitians as lactation counselors.	3 years	Primary care clinics. 17 sessions: 4 individual (third quarter) and 13 in groups (postpartum) at 1, 2, 4, 6, 9, 12, 15, 18, 21, 24, 27, 30 and 33 months.	Nutrition, breastfeeding and parenting.	The usual care.	Weight-for- height z-score and weight- for-age z-score, prevalence of obesity and excessive weight gain from birth to 2 years of age.	Mean weight-for age z-scores and growth trajectories were lower in the intervention group up to 2 years.
Ordway et al., 2018^21^	Socio-Ecological Model	Face-to-face. Social Worker with Master's Degree and Pediatric Nurse.	2 years	Home or place of convenience for the mother. Approximately 78 sessions (1 visit per week for 1 year, then fortnightly for 1 year).	Parent-child attachment, maternal reflective functioning and positive parenting behaviors.	The usual care.	Prevalence of overweight or obesity in children at 2 years of age.	More children in the intervention group had a healthy body mass index at 2 years of age.
Parat et al., 2018^22^	Not specified	Face-to-face. Physician, dietician, midwife.	27 weeks	Hospital. 6 sessions: 2 individual prenatal sessions (26 and 30 weeks of gestation and 4 group sessions (21, 28, 35 weeks of gestation, 2 months Postpartum).	Infant and maternal feeding	The usual care.	Infant weight gain from birth to 2 years of age.	Excessive weight gain during pregnancy was not significantly changed. Overweight in mothers and children 2 years after delivery was not prevented.
Fangupo 2015^23^ Taylor 2017^24^,2018^25^	Responsive Parenting	Face-to-face. Parents as Teachers (PAT): lactation consultant, trained researchers (nurses, dietitians, nutrition graduates). Sleep: nurse researcher; COMBO: includes both of the above.	18 months	Home and group. 8 sessions: 5 pre-delivery visits (3 face-to-face and 2 by telephone), postpartum 3 additional face-to-face contacts at 3, 9 and 18 months.	Food, activity and breastfeeding (FAB): Healthy eating, breastfeeding, physical activity.	Sleep or a combination (FAB plus Sleep)	Child's body mass index.	Food, activity and breastfeeding (FAB) had an unexpected long-term adverse effect (weight gain). A protective effect for obesity in those who received the 'sleep intervention'.
Fiks et al., 2017^26^	Social Learning Theory	Face to face. Facebook. Psychologist.	11 months	Hospital/Facebook. 2 face-to-face and follow-up via Facebook.	Infant feeding practices, sleep, positive parenting, maternal well-being.	Reminders for infant primary care visit	Weight-for-height z-score.	No significant differences in anthropometric parameters were found.
Thomson et al., 2018^27^	Social Cognitive Theory and Transtheoretical Model of Behavioral Change	Face-to-face. University educated women and trained in Parents as Teachers (PAT) curriculum.	18 months	Home. 17 sessions: 5 prenatal visits, 12 postnatal visits per month on average.	Parents as Teachers plus weight control, gestational and postnatal physical activity, breastfeeding, complementary feeding, tummy time and sedentary lifestyles	Parents as Teachers (PAT) training, parents' knowledge of child development, improving parenting practices, providing early detection of developmental delays.	Gestational weight gain. Weight status. Weight-for-height z-score, Weight- for-age z-score, Body Mass Index.	It was not effective in improving maternal weight and infant growth outcomes.
Wasser et al., 2020^28^	Not specified	Face-to-face. Peer educator.	15 months	Home. 6 sessions: 1 during pregnancy and at 3, 6, 9, 12 and 15 months after childbirth.	Mindful and responsive feeding practices.	Child safety guidance.	Weight-for-height z-score, Weight- for-age z-score, Body Mass Index.	It did not produce significant differences in infant growth.
Wen et al., 2012^29^; 2015^30^	Social Learning Theory and the Health Belief Model	Face-to-face. Community nurse.	2 years	Home. 8 sessions: (1 prenatal and at 1, 3, 5, 9, 12, 18 and 24 months)	Breastfeeding, complementary feeding, tummy time, active play, family nutrition, physical activity and sleep.	The usual care.	Body Mass Index.	It was effective in reducing the average Body Mass Index of two-year-olds.

### General characteristics and outcomes of effective interventions

Finally, as this is a scoping review, we did not carry out a comprehensive analysis of outcomes^31^; however, we found that effective interventions were delivered to pregnant women from the third quarter onwards. According to the components, these interventions were based on the major risk factors for childhood obesity, supported by cognitive-behavioral theories. Delivery was face-to-face, mainly by nurses, and the impact of the intervention was influential in maintaining or improving nutritional status. This is described in Table 3.

**Table 3:** General characteristics and outcomes of effective interventions to prevent overweight and obesity in the first 1000 days of life

Author/ Year	Intervention Name	Delivered by/Components	Environment and Sessions	Outcomes of Intervention vs. Control
Messito et al., 2020^20^	Starting Early Program	Registered dietitians as lactation consultants. Social Cognitive Theory, Nutrition, breastfeeding and parenting.	Primary care clinics. 17 sessions, 4 individual sessions in the third quarter and 13 group sessions from 1 to 33 months of age.	The infants in the intervention group had a lower weight-for age z-score at 18 months (0.49 vs. control 0.73, p = 0.04) and at 2 years (0.56 vs. 0.81, p = 0.03).
Ordway et al., 2018^21^	Minding the Baby	Social workers and pediatric nurses. Socio-ecological model. Parent-child attachment, maternal reflective functioning and positive parenting behaviors.	Home visits. Weekly pediatric sessions from the third quarter of pregnancy until the child's first birthday, and then every two weeks until the child's second birthday.	The rate of obesity was significantly higher (p = 0.01) in the control group (19.70%) compared to the intervention group (3.30%) at 2 years (odds ratio = 0.32, 95% CI [0.13-0.78] p = 0.01).
Wen et al., 2012; 2015^29^^, ^^30^	Healthy Beginnings	Community nurses. Social Learning Theory and Health Beliefs. Breastfeeding, complementary feeding, tummy time, active play, family nutrition, physical activity and sleep.	Home visits. One visit in the prenatal period and 7 visits at 1-24 months of age.	The mean BMI was significantly lower in the intervention group (16.53) than in the control group (16.82), with a difference of 0.29 (95% CI [-0.55-0.02] p = 0.04).

## Discussion

The aim of this study was to identify and characterize interventions delivered during the first 1000 days of life for the prevention of childhood obesity. We identified 10 interventions reported in 14 articles between 2012 and 2022.

We found that the development and implementation of interventions are concentrated in high- income countries, in line with other systematic reviews^11^^, ^^12^^, ^^32^, and that while in the past overweight was almost exclusively a problem of high-income countries, prevalence patterns have now changed, with low-and middle-income countries accounting for three-quarters of childhood overweight worldwide^2^^, ^^33^. Policies focusing on the prevention of childhood obesity in the first 1000 days have recently been implemented in Latin America^34^ and publications from this region with positive results are expected soon.

More than half of the interventions included racial/ethnic subgroups in their study populations, such as Latinos and African Americans, who are considered to be groups with a high prevalence of risk factors for childhood obesity^35^^, ^^36^; however, they did not include cultural or environmental aspects beyond the vulnerability or ethnicity of the population, which is seen as an area of opportunity. This highlights the need for future interventions to consider the socioeconomic status, environment and culture in which children and families live, as these variables may influence weight-related behaviors, as do dietary practices and physical activity^37^^, ^^38^.

Regarding the start of implementation, most interventions were initiated in the third quarter of pregnancy; however, evidence suggests that ideally they should be initiated before conception, but if this is not possible, they should be initiated from the first quarter of pregnancy and continued until the child is two years old^39^. As previously mentioned, it was found that none of the interventions included preconception care, but they did include follow-up for two years or more, which aligns with the scientific evidence reported in previous systematic reviews^11^^, ^^12^^, ^^40^. Preventive interventions delivered at these stages may prepare the mother-to-be to reduce risk factors for childhood obesity, and there is, therefore, a need to continue to research this period^8^^, ^^9^^, ^^11^^, ^^38^.

This scoping review found three interventions with statistically significant results: StEP^20^, Minding the Baby^21^ and Healthy Beginnings^29^. Key features of these interventions were that they were multi component and targeted the main risk factors for obesity in early childhood; they also used theoretical models that focus on how perceptions, beliefs and thoughts influence behavior. This allows for a coherent, evidence-based structure that facilitates the understanding of health determinants, the prediction and evaluation of out comes,and the reproducibility and generalizability of interventions^6^^, ^^9^^, ^^41^.

The delivery method for these three interventions was face-to-face home visits, with a minimum follow-up of 2 years. Evidence suggests that, beyond the provision of information, this individualized face-to-face support builds a relationship of trust between the facilitator and the mother, promoting favorable outcomes in terms of improvements in developmental indicators, infant feeding practices and parenting, all in disadvantaged families^42^^, ^^43^.

Likewise, it was seen that the use of technology was incorporated into the interventions, the most common being telephone calls, text messages and computer use. The use of technological resources to promote health is an opportunity to bring educational strategies to the population^44^. In recent years, the number of people using these tools on a daily basis has increased, providing an opportunity for educators and researchers to reduce the cost of implementing obesity prevention interventions, thus reducing the gap in access to health services^45^^, ^^46^.

Cultural adaptations are considered necessary to improve identified outcomes^47^, and the use of technology and interventions that take into account multifactorial aspects such as culture, dietary practices and physical activity has significant effects on participants' weight^32^^, ^^48^.

Limitations include the fact that, by its nature, this scoping review did not include a quality assessment of the reviewed studies or an in-depth review of the statistical results, which may limit the generalizability of the findings. Strengths include that the design allowed for a systematic synthesis of research characteristics and findings, which could contribute to health professionals' decision making in preventing of childhood obesity.

## Conclusion

It is encouraging that from the ten selected interventions, three were found to be effective in preventing childhood overweight and obesity in the first 1000 days of life. The characteristics summarized in terms of study population, intervention design and outcomes of effective interventions provide evidence that can be used to determine whether these interventions will work in other contexts, such as low- and middle-income countries and in groups vulnerable to developing childhood obesity.

In addition, the use of cultural adaptation processes could be a useful strategy to reduce development and implementation time and optimize resources. Ensuring that interventions cover all of the above aspects will result in better prevention of childhood obesity.
